# Robotic-Assisted Bronchoscopy and Pulmonary Nodules Due to Atypical Infections in Coccidioidomycosis Endemic Area

**DOI:** 10.1016/j.chpulm.2026.100263

**Published:** 2026-05-02

**Authors:** Robert Lee, Selina Deanda, Wali Zaidi, Paul Kang, Kenneth Sakata, Carmold Murray, Merica Vorachitti, Stephen Bruce, Deepika Razia, Ariba Moin, Devika Sindu, Ali Imran Saeed

**Affiliations:** a UC Health Parkview Medical Center, Pueblo, CO; b UT Houston McGovern Medical School, Houston, TX; c Creighton University, Omaha, NE; d Mayo Clinic, Scottsdale, AZ; e University of Wisconsin Hospital and Clinic, Madison, WI; f Mercy St Vincent’s Medical Center, Toledo, OH; g Norton Thoracic Institute, Saint Josephs Hospital and Medical Center, Phoenix, AZ

**Keywords:** atypical infection, benign pulmonary nodule, bronchoalveolar lavage, procedural complication, robotic bronchoscopy

## Abstract

**Background:**

Although robotic-assisted bronchoscopy (RAB) primarily aims to diagnose early stage lung cancer, many pulmonary nodules stem from benign causes, including atypical infections (ie, *Coccidioides* species, *Aspergillus* species, nontuberculous mycobacteria). Data on prevalence of atypical infections in patients undergoing RAB with biopsy of pulmonary nodules are limited.

**Research Question:**

What is the prevalence of atypical and bacterial infections in patients undergoing robotic-assisted bronchoscopy with biopsy of pulmonary nodules in a coccidioidomycosis endemic area?

**Study Design and Methods:**

This single-center, retrospective study examined 526 patients who underwent RAB with a shape-sensing platform at our institution from June 2021 through December 2023.

**Results:**

Of the 526 patients, 261 (49.6%) had malignancy without infection, 107 (20.3%) had cultures positive for infection without malignancy, and 35 (6.65%) were diagnosed with both malignancy and a coexisting bacterial and/or atypical infection. *Coccidioides* serology (IgG) or clinical history was significantly more prevalent in the infection without malignancy group (32.7%) and the malignancy with infection group (42.9%) compared with the malignancy-only group (0%; *P* < .001). Complication rates (pneumothorax, bleeding, and hospitalization) did not differ significantly between groups. In a subgroup analysis, 73 patients (13.9%) with granulomatous inflammation on pathology had higher rates of positive fungal (*P* = .002), *Aspergillus* (*P* = .042), and acid-fast bacilli (*P* < .001) cultures than patients in the nongranuloma group. Positive cultures of acid-fast bacilli, *Coccidioides* species, or *Aspergillus* species also had granulomatous inflammation in 76%, 29%, and 29% of cases, respectively. Fifty-two patients had > 1 positive culture result.

**Interpretation:**

This study highlights the importance of obtaining bronchoscopy cultures in patients undergoing RAB, especially when rapid on-site evaluation of the pulmonary nodule biopsy does not reveal malignancy.

**Take-Home Points**

**Study Question:** What is the prevalence of atypical and bacterial infections in patients undergoing robotic-assisted bronchoscopy with biopsy of pulmonary nodules in a coccidioidomycosis endemic area?

**Results:** A significant percentage of patients (20.3%) undergoing robotic bronchoscopy with biopsy of pulmonary nodules were found to have infection on culture as the primary diagnosis; coexisting malignancy and infection was in 6.65% of patients.

**Interpretation:** This study highlights the importance of obtaining bronchoscopy cultures in patients undergoing robotic-assisted bronchoscopy, especially when rapid on-site evaluation of the pulmonary nodule biopsy does not reveal malignancy.

Navigational bronchoscopy for biopsy of peripheral pulmonary nodules offers a diagnostic yield comparable with that of percutaneous CT scan-guided needle biopsy, with a significantly lower complication rate.^1^ Advancements, particularly the development of robotic platforms, have consistently demonstrated higher diagnostic yields with low complication rates.^2^ Although the primary goal of robotic-assisted bronchoscopy (RAB) is the diagnosis of early stage lung cancer, a substantial number of pulmonary nodules are attributed to benign causes, including bacterial and atypical infections. However, studies detailing the prevalence of atypical infections (eg, *Coccidioides* species, *Aspergillus* species, nontuberculous mycobacteria [NTM]) presenting as pulmonary nodules in patients undergoing RAB are limited.

Radiographic indicators such as smooth nodule borders, surrounding satellite nodules, tree-in-bud nodules, and a halo of ground-glass opacification suggest an infectious etiology of pulmonary nodules.^3^^,^^4^ Current management guidelines typically recommend repeat imaging at appropriate intervals to monitor the stability or resolution of the nodule.^5^ Nevertheless, managing a pulmonary nodule with concerning radiographic appearance or a patient with a history of tobacco abuse and/or a personal or strong family history of cancer presents a challenge to the watch-and-wait approach.

In this study, we aimed to determine the prevalence of atypical infections in patients undergoing RAB with biopsy of pulmonary nodules in coccidioidomycosis endemic area. We also compared demographic and clinical characteristics and procedural complication rates between 3 patient groups based on diagnosis: malignancy, infection, or malignancy and infection. We performed a subanalysis of infection types in patients with granulomatous inflammation of pulmonary nodules. Finally, we performed exploratory analyses to compare patients with a benign diagnosis (with or without infection) with those with a cancer diagnosis. Nodule size and location were described for patients diagnosed with infection, malignancy, or neither, and infection types were detailed using frequencies and percentages for types of organisms.

## Study Design and Methods

### Patient Population

This single-center, retrospective study, approved by the institutional review board of St. Joseph’s Hospital and Medical Center (No. PHX-24-500-292-71-18) for data collection and analysis with a waiver of patient consent because of the study design, included patients who underwent robotic bronchoscopy from June 2021 through December 2023. Two board-certified interventional pulmonologists using a shape-sensing platform performed the RAB procedures. Seven internal medicine residents and 2 research fellows were educated on robotic bronchoscopy, the study hypotheses, and efficient collection of data using a standardized data sheet (e-Appendix 1) to capture each variable. To ensure consistency and reduce errors, a data audit was also conducted.

### Covariates

Demographic variables evaluated in the study included age, sex, and BMI; race/ethnicity; and smoking status. Smoking status encompassed people who currently smoke and people who formerly smoked, with associated pack-years and years since cessation. The study also considered a history or serologic evidence of coccidioidomycosis. Sex, smoking status, and coccidioidomycosis history/serology were all treated as binary (yes/no) variables. Race/ethnicity was categorized into White, Hispanic, Black, Native American, or other (southeast asian or those who prefer not to answer).

The biopsy site was recorded as right upper lobe, right middle lobe, right lower lobe, left upper lobe, lingula, left lower lobe, and other. Instances when 2 nodules were biopsied in the same lobe were recorded. Nodule size was grouped according to its maximum diameter as follows: 0 to 5, 6 to 10, 11 to 20, 21 to 30, and > 31 mm. The nodule’s lung position was classified as inner, middle, or outer third. Biopsy results were reported as benign, malignant, or nondiagnostic based on a Delphi consensus statement.^6^ Additionally, positive infection status was determined if cultures revealed *Coccidioides* species, *Aspergillus* species, acid-fast bacteria (AFB), or other fungal pathogens.

Key aspects of the procedural outcomes included the use of intraoperative CT scan and the placement of fiducial markers. Instrumentation was categorized into cryoprobe, needle, forceps, or brush. Complications, including bleeding, pneumothorax, chest tube placement, and hospitalization, were recorded as binary variables. The estimated blood loss was noted for bleeding complications. Procedure duration was measured by both total room time and bronchoscopy time. Postprocedural pneumothorax was identified through procedural documentation, postbronchoscopy chest radiographs, or hospitalization, specifically for pneumothorax. Intraoperative bleeding complications were determined by reviewing interventional pulmonology notes, specifically looking for cases that required the administration of cold saline with tranexamic acid, procedure interruption, or hospitalization because of hemorrhage.

Rapid on-site evaluation (ROSE) by the staff pathologist guided placement of a fiducial marker if the preliminary report suggested malignancy or atypical cells. The decision to place a fiducial marker when ROSE was nondiagnostic rested with the interventional pulmonary consultant, taking into consideration the patient’s baseline risk of malignancy and the need to document the location of transbronchial biopsy in relation to the nodule that yielded the nondiagnostic ROSE.

### Statistical Analysis

Patient demographics, nodule characteristics, and clinical outcomes were summarized using medians and interquartile ranges for continuous variables, given their nonnormal distributions, and frequencies and percentages for categorical variables. To compare continuous and ordinal variables across groups, the Kruskal-Wallis test was used. Categorical variables were compared using the χ^2^ or Fisher exact tests, as appropriate. For statistically significant overall *P* values, Bonferroni-corrected pairwise comparisons were conducted (*P* < .016, derived from 0.05 divided by the number of comparisons). All statistical analyses were performed using STATA version 18 (StataCorp). All *P* values were 2-sided, with *P* < .05 considered statistically significant.

## Results

A total of 526 patients met the inclusion criteria and were categorized into 3 diagnostic groups: malignancy (n = 261), infection (n = 107), or both malignancy and infection (n = 35) for the primary analysis (Table 1). Patients with benign nodules without infection (n = 65) are subsequently discussed. Patients with nondiagnostic results (n = 58) were followed for up to 2 years (e-Table 1).

**Table 1:** Demographic Characteristics of the Patient Groups

Variable	All Patients^a^ (N = 526)	Malignancy Without Infection (n = 261; 49.6%)	Infection Without Malignancy (n = 107; 20.3%)	Malignancy With Infection (n = 35; 6.65%)	*P* Value
Age, y	69 (61-76)	71 (65-77)	68 (57-73)^b^	70 (62-75)	.003
Sex					.50
Female	273 (51.9)	132 (50.6)	52 (48.6)	21 (60.0)	
Male	253 (48.1)	129 (49.4)	55 (51.4)	14 (40.0)	
BMI, kg/m^2^	26.6 (22.8-31.1)	26.7 (22.9-30.2)	25.3 (21.7-31.4)	25.4 (21.5-29.4)	.49
Race					.095
White	424 (80.6)	220 (84.3)	80 (74.8)	31 (88.6)	
Hispanic	38 (7.22)	16 (6.13)	6 (5.61)	2 (5.71)	
Black	22 (4.18)	10 (3.83)	5 (4.67)	2 (5.71)	
Native American	4 (0.76)	1 (0.38)	2 (1.87)	0 (0.0)	
Other	38 (7.22)	14 (5.36)	14 (13.1)	0 (0.0)	
Smoking status	136 (25.9)	78 (29.9)	25 (23.4)	9 (25.7)	.45
Pack-years	25 (0-42)	30.5 (5-50)	17 (0-39)^b^	22 (15-45)	.002
Years since quitting	9 (2-23)	11.5 (3-25)	8 (2.5-22)	7.5 (1.5-32.5)	.91
Cocci history or positive serology	57 (10.8)	0 (0.0)	35 (32.7)^b^	15 (42.9)^c^	< .001
Multiple biopsies	150 (25.5)	80 (30.7)	25 (23.4)	8 (22.9)	.30
Bilateral biopsies (n = 150)	82 (54.7)	42 (52.5)	13 (52.0)	7 (87.5)	.17

Data are presented as median (interquartile range [Q1-Q3]), No. (%), or as otherwise indicated. Continuous variables were compared using the Kruskal-Wallis test. Categorical variables were compared using the χ^2^ analysis or Fisher exact test. Cocci = coccidioidomycosis.

^d^Statistical difference between group 2 and group 3 after Bonferroni correction (*P* < .016).

a Patients with no malignancy and no infection (n = 65) and those with nondiagnostic results (n = 58) are not shown.

b Statistical difference between group 1 and group 2 after Bonferroni correction (*P* < .016).

c Statistical difference between group 1 and group 3 after Bonferroni Correction (*P* < .016).

Significant differences were observed across several variables (Table 1). The median age was significantly higher in the malignancy-only group (71 years of age; interquartile range, 65-77) than in the infection-only group (68 years of age; interquartile range, 57-73; *P* = .003; Bonferroni *P* < .016). Similarly, smoking exposure (pack-years) was greater in patients with only malignancy than in those with only infection (Bonferroni *P* < .016). No significant differences were found for sex, BMI, or smoking status. Although race distribution varied between groups, the differences did not reach statistical significance (*P* = .095). Overall, most patients were White (80.6%).

*Coccidioides* serology or clinical history of coccidioidomycosis was significantly more prevalent in the infection-only group (32.7%) and the malignancy with infection group (42.9%) than the malignancy-only group (0%; *P* < .001). Finally, procedure-related variables such as multiple biopsies and bilateral sampling showed no significant differences between the groups.

### Procedural Outcomes and Complication Rates

Three diagnostic groups were analyzed for procedural characteristics and complication rates: infection only (n = 107), malignancy only (n = 261), and benign without infection (n = 65) (Table 2). Intraoperative CT scan use was similar across groups (*P* = .053), ranging from 40.2% in the malignancy group to 56.9% in the benign group. Fiducial marker placement was significantly more common in the malignancy (31.8%) group than in the infection-only group (10.3%) or benign without infection group (15.4%; Bonferroni *P* < .016), consistent with its use in pretreatment localization for cancer therapy.

**Table 2:** Bleeding and Pneumothorax Rates in Patients With a Diagnosis of Infection, Malignancy, or Benign Lesion Without Infection

Variable	Infection (No Malignancy) (n = 107; 24.3%)	Malignancy (No Infection) (n = 261; 59.2%)	Benign (No Infections) (n = 65; 14.7%)	*P* Value
Intraoperative CT scan, yes	46 (42.9)	105 (40.2)	37 (56.9)	.053
Fiducial marker, yes	11 (10.3)	83 (31.8)^a^	10 (15.4)^b^	< .001
Instrument				
1.1-mm cryoprobe	100 (93.5)	247 (94.6)	61 (93.8)	.84
Needle	44 (41.1)	93 (35.6)	20 (30.8)	.31
Forceps	3 (2.80)	3 (1.15)	4 (6.15)	.049
Brush	21 (19.6)	77 (29.5)	9 (13.9)^b^	.012
Complications				
Bleeding	1 (0.93)	6 (2.30)	1 (1.54)	.88
Blood volume, mL	100 (100-100)	87.5 (50-100)	30 (30-30)	.26
Pneumothorax	1 (0.93)	5 (1.92)	4 (6.15)	.096
Chest tube	0 (0.0)	3 (1.15)	3 (4.62)	.056
Hospitalization	2 (1.87)	7 (2.68)	4 (6.15)	.29
Procedure total room time	66 (55-79)	71 (56-84)	68 (53-84)	.43
Bronchoscopy time	41 (30-55)	50 (34-62)	45 (34-62)	.11

Data are presented as median (interquartile range [Q1-Q3]), No. (%), or as otherwise indicated. Continuous variables were compared using the Kruskal-Wallis test. Categorical variables were compared using the χ^2^ analysis or Fisher exact test.

^c^Statistical difference between group 1 and group 3 after Bonferroni correction (*P* < .016).

a Statistical difference between group 1 and group 2 after Bonferroni correction (*P* < .016).

b Statistical difference between group 2 and group 3 after Bonferroni correction (*P* < .016).

Instrument use was comparable across groups. A cryoprobe (1.1 mm) was used in > 93% of cases regardless of diagnosis. Needle usage ranged from 30.8% to 41.1% with no significant differences between groups (*P* = .31). Forceps were used more frequently in cases with benign lesions (6.15%) than in those with malignant (1.15%) or infectious lesions (2.8%; *P* = .049). Brushings were performed more often in malignant cases (29.5%) than benign cases (13.9%; *P* = .012), suggesting a preference for cytologic sampling in suspected cancer lesions.

Complication rates were low across all groups. Bleeding ranged from 0.93% in the infection group to 2.3% in the malignancy group (*P* = .88), with the median estimated blood loss similar across groups (*P* = .26). Pneumothorax and chest tube placement were slightly more common in the benign group (6.15% and 4.62%, respectively), but these differences did not reach statistical significance (*P* = .096 and *P* = .056, respectively). Hospitalization rates because of procedural complications were low and similar across all groups (*P* = .29). Total procedure room time and bronchoscopy time were also similar (*P* = .43 and *P* = .11, respectively).

Table 3 compares infectious testing results between patients with histologic evidence of granuloma (n = 73; 13.9%) and those without (n = 453; 86.1%). A strong association was observed between granulomatous inflammation and fungal infection because fungal culture positivity was significantly higher in the granuloma group (23.3%) than the nongranuloma group (9.71%; *P* = .002). This link was further supported by the more frequent isolation of *Aspergillus* species in patients with granulomas than those without (10.9% vs 4.42%; *P* = .042). Consistent with the known association between granulomas and mycobacterial infections, AFB cultures were significantly more likely to be positive in the granuloma group (17.8% vs 0.88%; *P* < .001). Although bacteria and other fungal organisms were cultured more frequently in patients with granulomas, these differences were not statistically significant (*P* = .096 and *P* = .21, respectively). *Coccidioides* detection (by serology, pathology, or culture) was higher in the granuloma group, but this difference did not reach statistical significance (all *P* > .05).

**Table 3:** Descriptive Analysis of Serology, Pathology, and Cultures by Granuloma Status

Infections	Overall (N = 526)	No Granuloma (n = 453; 86.1%)	Granuloma (n = 73; 13.9%)	*P* Value
Positive Cocci serology	57 (10.8)	46 (10.2)	11 (15.1)	.22
Cocci on pathology	20 (3.80)	16 (3.53)	4 (5.48)	.50
Positive Cocci culture	14 (2.66)	10 (2.21)	4 (5.48)	.12
Fungal culture	61 (11.6)	44 (9.71)	17 (23.3)	.002
*Aspergillus* culture	28 (5.32)	20 (4.42)	8 (10.9)	.042
Other fungus	22 (4.18)	17 (3.75)	5 (6.85)	.21
AFB culture	17 (3.23)	4 (0.88)	13 (17.8)	< .001
Bacteria culture	55 (10.5)	43 (9.49)	12 (16.4)	.096

Data are presented as No. (%) or as otherwise indicated. Categorical variables were compared using the χ^2^ analysis or Fisher exact test. AFB = acid-fast bacteria; Cocci = *Coccidioides*.

Granulomas were observed in a notable proportion of patients across various infectious categories. Among the patients with positive *Coccidioides* serology (n = 57), 11 (19.3%) presented with granulomas. This proportion rose to 20.0% when *Coccidioides* species was identified on pathology and 28.6% with positive *Coccidioides* cultures. For patients with any positive fungal culture (n = 61), granulomas were seen in 27.9% of cases, specifically in 28.6% with *Aspergillus* species and 22.7% with other fungal organisms. The highest proportion of granulomas (76.5%) was found in patients with positive AFB cultures. In contrast, bacterial culture positivity showed a lower association with granulomas (21.8%) (Table 4).

**Table 4:** Presence of Granuloma Within Each Positive Infection

Infection	Total No. of Positive Infections	Granuloma, No. (%)
Cocci in serology	57	11 (19.3)
Cocci on pathology	20	4 (20.0)
Cocci in culture	14	4 (28.6)
Fungal culture	61	17 (27.9)
*Aspergillus* culture	28	8 (28.6)
Other fungus	22	5 (22.7)
AFB culture	17	13 (76.5)
Bacteria culture	55	12 (21.8)

AFB = acid-fast bacteria; Cocci = *Coccidioides*.

The prevalence by infection type in patients with multiple infections is presented in Table 5. Although the percentage of positive *Coccidioides* cultures remained steady as the number of total infections increased, the percentage of *Aspergillus* species increased significantly from 0% to 59.1% as the number of infections rose from 1 to 3 (*P* < .001). Similar trends were noted for other fungal infections (*P* < .001). The percentage of positive bacterial infections was lowest in patients with 2 total infections.

**Table 5:** Cultured Organisms in Relation to the Total Number of Infections

Infections	1 Infection (n = 57)	2 Infections (n = 30)	3 Infections (n = 22)	*P* Value
Cocci in culture	10 (17.5)	4 (13.3)	0 (0.0)	.079
*Aspergillus* culture	0 (0.0)	15 (50.0)	13 (59.1)	< .001
Other fungus	0 (0.0)	12 (40.0)	10 (45.5)	< .001
AFB culture	13 (22.8)	1 (3.33)	3 (13.6)	.054
Bacteria culture	34 (59.7)	3 (10.0)	18 (81.8)	< .001

Data are presented as No. (%) or as otherwise indicated. Percentages may add up to > 100 because of multiple infections. No infection: n = 417 (not shown). AFB = acid-fast bacteria; Cocci = *Coccidioides*.

### Exploratory Analysis

Demographic and clinical characteristics, categorized by final diagnosis (benign without infection: n = 65; benign with infection: n = 107; malignant: n = 296), are presented in e-Table 2. Patients in the malignant group were older than those in the benign with infection and benign without infection groups (Bonferroni *P* < .016 for both). Smoking exposure (pack-years) was notably higher in the malignant group than the benign groups (*P* < .001). *Coccidioides* serology or clinical history was considerably more prevalent in the infection group (32.7%) than in the malignant (5.07%) or benign noninfectious groups (0%; *P* < .001). Sex, BMI, race, smoking status, years since quitting, frequency of multiple biopsies, and bilateral procedures did not show significant differences.

Nodule size and location varied significantly by diagnosis (*P* = .026 and *P* = .016, respectively), as presented in e-Table 3. Malignant nodules were more frequently centrally located, with 21.7% in the inner third of the lung and 31.8% in the middle third of the lung. Conversely, infectious nodules were primarily peripheral, with 59.0% found in the outer third of the lung.

Regarding size, larger nodules (> 30 mm) appeared more often in the infection (18.1%) and malignancy (14.7%) groups compared with the benign group (6.5%). Although benign lesions showed a wider distribution across size categories, most nodules in both the infection and malignancy groups measured 11 to 20 mm (40.9% and 41.7%, respectively).

e-Table 4 details the relationships between identified infectious organisms and other microbiological findings. Patients with positive *Coccidioides* serology (n = 57) showed a significantly higher likelihood of coccidioidomycosis being identified on pathology (11.3% vs 2.8%; *P* < .01) and culture (17.5% vs 0.85%; *P* < .001) and increased rates of fungal culture positivity (29.8% vs 9.4%; *P* < .001). Similarly, among patients with positive fungal cultures (n = 61), there was a notable increase in the detection of coccidioidomycosis on pathology (11.5%; *P* < .001) and culture (22.9%; *P* < .01). These patients also exhibited higher rates of *Aspergillus* species (45.9%; *P* < .001), other fungal organisms (36.1%; *P* < .001), and bacterial coinfection (32.8%; *P* < .001).

Positive bacterial cultures were linked to higher rates of fungal (36.4%; *P* < .001), *Aspergillus* (16.4%; *P* < .01), and other fungal (16.4%; *P* < .01) growth. Although not statistically significant, fungal coinfection was more common in patients with AFB culture positivity (n = 17) (17.6% vs 11.4%). Importantly, all 28 patients with positive *Aspergillus* cultures also had positive fungal cultures (100%; *P* < .001) and were more prone to concurrent bacterial infections (32.1% vs 9.2%; *P* < .01).

Forty-six nodules with concurrent malignancy and infection were found in 35 patients (e-Table 5). Most of the nodules were located within the outer third of the lung (56.5%). Nodules from 11 to 20 mm in size were the most common (43.5%), followed by those > 31 mm (21.7%). Adenocarcinoma was diagnosed in final pathology reports in 22 of 35 patients (62.9%), whereas squamous cell carcinoma was diagnosed in 6 patients (17.1%). Concerning infections, positive bacteria (40.0%) and fungal cultures (22.9%) were the most common.

## Discussion

Recent strides in navigational bronchoscopy, particularly the advent of RAB platforms, have enabled precise navigation and biopsy of peripheral pulmonary nodules. The integration of intraoperative CT scans with RAB facilitates real-time localization and adjustment of the robotic catheter tip. This not only enhances diagnostic yield but also reduces complication rates, through a clearer understanding of the pleural edge and surrounding blood vessels. Although the primary objective of RAB is the diagnosis of early stage lung cancer, a considerable number of pulmonary nodules are attributed to infectious etiologies. The exact prevalence of atypical infections diagnosed via robotic bronchoscopy remains ill-defined, varying with endemic organisms and patient demographics. Pulmonary nodules resulting from atypical infections often exhibit rounded smooth borders, smaller adjacent satellite nodules, and a halo of ground-glass opacification.^3^^,^^4^

Pulmonary nodule risk calculators and Fleischner guidelines,^5^ alongside a multidisciplinary approach, are routinely used to determine appropriate follow-up CT scan intervals. Patients at high risk, including those with a personal history of smoking or cancer, a family history of lung cancer, or exposure to asbestos or radon, typically necessitate more frequent imaging and/or biopsy.

RAB allows for the simultaneous biopsy of multiple pulmonary nodules with a low complication rate. ROSE of the cytopathology of biopsy samples can guide the proceduralist in performing tissue culture and bronchoalveolar lavage (BAL), especially when ROSE suggests granuloma, fungal organisms, or nondiagnostic biopsy results.

Microbiome of the lungs includes microorganisms (ie, bacteria, fungi, viruses) that colonize the lining of airways and associated molecules, including genes, genome, secreted factors, and metabolites. There is no clear association of lung cancer development with microbial dysbiosis.^7^ A study by Yuan et al^8^ revealed an altered microbiome of pulmonary nodules with a diagnosis of pulmonary adenocarcinoma. Dedicated BAL collected just before lobectomy revealed *Streptococcus mitis*, *Burkholderia oklahomensis*, and *Burkholderia latens*. Microbiome alterations because of emphysema, interstitial lung disease, bronchiectasis, and so forth may explain a higher probability of microbial colonization because of the use of steroids, immunosuppression, and repeated courses of antibiotics.^9^^,^^10^ We collected dedicated BAL using the robotic hollow catheter after pulmonary nodule biopsy with 40 to 60 mL of sterile water, instilled and collected with a 20-mL syringe. This approach allows for further understanding of the pulmonary nodule microbiome, especially in coccidioidomycosis endemic areas, and the need for development of a standardized diagnostic protocol to streamline infectious workup for a pulmonary nodule. Our current study involved a meticulous retrospective review to gain insights into the prevalence of atypical infections in patients undergoing RAB. Of the 526 patients, 107 (20.3%) were identified with pulmonary nodules because of atypical infections. RAB revealed coexisting infection with malignancy in 35 patients (6.65%). Complication rates, including pneumothorax, chest tube placement, hospitalization, and bleeding, were similar across patients diagnosed with malignancy, infection, or benign etiologies. Seventy-three patients (13.9%) were found to have granuloma on pathology, and a clinically significant association was observed between granulomatous inflammation and positive fungal culture (23.3%), *Aspergillus* cultures (10.9%), and AFB culture (17.8%) when compared with the nongranuloma group. *Coccidioides* serology (IgG) and history were positive in 57 patients (20.8%), and pathology with granuloma showed the highest concordance with culture for NTM infections (76%), followed by *Coccidioides* and *Aspergillus* culture (28% each). Interestingly, multiple overlapping infections were also found in BAL and tissue cultures: 30 patients had simultaneous culture growth of 2 organisms and 22 patients had 3 simultaneous organisms.

Coccidioidomycosis, a fungal infection endemic to the Southwestern US, poses a diagnostic challenge during lung cancer screening.^11^ The precise incidence of false positives attributed to coccidioidomycosis in patients undergoing low-dose CT scans for lung cancer screening in endemic regions is not yet established. Pulmonary coccidioidomycosis can manifest as lung masses with irregular borders and spiculation or as thick-walled cavitary pulmonary nodules, making differentiation from malignancy even more difficult.^12^ Furthermore, the standardized uptake values of pulmonary nodules caused by coccidioidomycosis can vary widely on PET scans, making infection and malignancy indistinguishable.^13^^,^^14^

For patients exhibiting symptoms of acute infection, quantitative immune diffusion and complement fixation tests are the most frequently used diagnostic methods.^15^^,^^16^ However, the utility of *Coccidioides* serology in diagnosing latent infection presenting as a pulmonary nodule remains uncertain. In the current review of 526 patients, only 57 patients (10.8%) had a current or previous positive *Coccidioides* IgG result; however, none of the patients in the malignancy-only group had elevated *Coccidioides* serology, whereas 32.7% of the infection group and 42.9% of the malignancy with infection group did have an elevated IgG level.

*Aspergillus* species are widespread in the environment. Clinical disease typically arises in individuals with compromised immune systems, genetic predispositions, or preexisting lung damage.^17^ Symptomatic *Aspergillus* lung infection is very rare in immunocompetent individuals.^18^ Patients with pulmonary aspergillosis can present with single or multiple pulmonary nodules, which are often challenging to diagnose clinically or serologically, and therefore may require biopsy or surgical excision.^19^^,^^20^

Kang et al^21^ reported on 11 immunocompetent patients with a histologic diagnosis of pulmonary aspergillosis manifesting as pulmonary nodules; 3 of these patients showed evidence of cavitation within the nodules on CT imaging. Yoon et al^22^ described a series of 7 patients from Korea who had biopsy-proven pulmonary aspergillosis without immunosuppression or underlying lung disease. In the current study, 28 patients were diagnosed with *Aspergillus* infection via culture. Of these patients, 8 (28%) showed granulomatous inflammation on pathology, 15 had 1 coexisting infection, and 13 had 2 coexisting infections in addition to *Aspergillus* species, raising suspicion for colonization rather than true infection.

Nontuberculous mycobacterial infections are frequently observed in individuals with preexisting lung conditions and compromised immune systems.^23^ Common clinical manifestations include shortness of breath, cough, fatigue, weight loss, night sweats, and chest congestion. Chest CT scan typically reveals 3 primary radiographic abnormalities associated with NTM infection: cavitary nodules, clustered nodules, and bronchiectasis.^6^^,^^24^

The American Thoracic Society,^25^ along with others, has established specific diagnostic criteria for NTM. These biopsy criteria mandate histologic features such as granulomatous inflammation or a positive AFB stain, along with ≥ 1 sputum or bronchial wash cultures positive for NTM.^26^ However, the medical literature provides limited information regarding the prevalence of NTM detected in bronchial wash cultures from patients undergoing RAB. Pathak et al,^27^ for instance, reported only 1 case of NTM infection in a cohort of 51 patients who underwent RAB. In our current study, we identified 17 patients with BAL positive for AFB culture, with granuloma formation on pathology demonstrating the strongest association (76.5%) with NTM infection.

The current study reveals a notable prevalence of multiple simultaneous organisms on BAL and tissue culture: 30 patients with simultaneous culture growth of 2 organisms and 22 patients with 3 simultaneous organisms. This observation prompts further inquiry into whether these instances represent simultaneous colonization or true infections. Fifty-eight patients undergoing robotic bronchoscopy were found to have negative cultures and a nondiagnostic result per Delphi criteria.^28^

RAB has transformed the diagnostic process for peripheral pulmonary nodule biopsies. Although 30% to 50% of these biopsies reveal a benign etiology, the prevalence of atypical infections identified during RAB with subsegmental bronchial washes, which are typically collected after the biopsy and before bronchoscope withdrawal, is unknown.

To our knowledge, this study is 1 of the largest retrospective reviews on the prevalence of atypical infections and their pathologic correlates. A novel finding is the 6.6% incidence of infection in patients with a biopsy-proven malignancy. Granulomatous inflammation was the most common pathologic finding in patients with atypical infections. Notably, NTM infections, followed by atypical fungal infections, showed the highest concordance with pathology demonstrating granulomatous inflammation. The culture of multiple simultaneous organisms raises concerns about colonization, necessitating expert consultation for treatment decisions.

A key limitation of the current study stems from the lack of standardized collection methods for BAL and treatment. BAL and tissue culture were often collected after preliminary pathology reports indicated an absence of malignancy (ie, nondiagnostic results, presence of granuloma). In cases where multiple microorganisms grew on BAL, it was difficult to distinguish between colonization and true infection needing appropriate treatment. When an appropriate treatment was offered, especially in symptomatic patients, in a significant number of patients, the culture results were forwarded to a referring consultant for management. Furthermore, the study center’s location in a coccidioidomycosis-endemic area likely contributed to a higher observed prevalence of atypical infections.

## Interpretation

Our results show that significant portion of pulmonary nodules in patients undergoing RAB are caused by benign or infectious etiology. A standardized protocol for the collection of BAL and culture of tissue biopsies, particularly when rapid on-site pathology evaluation reveals granulomatous inflammation or is nondiagnostic, should be established.

## Funding/Support

The authors have reported to *CHEST Pulmonary* that no funding was received for this study.

## Financial/Nonfinancial Disclosures

The authors have reported to *CHEST Pulmonary* the following: A. I. S. is a robotic bronchoscopy proctor for Intuitive Surgical and an advisor for drug and device development for Maverick Medical; there is no direct conflict of interest. None declared (R. L., S. D., W. Z., P. K., K. S., C. M., M. V., S. B., D. R., A. M., and D. S.).

## Supplementary Data

e-Online Data
