# Supplementary material for: Robotic-Assisted Bronchoscopy and Pulmonary Nodules Due to Atypical Infections in Coccidioidomycosis Endemic Area
Source: CHEST Pulm. 2026 May 2;4(3):100263. doi: 10.1016/j.chpulm.2026.100263 (PMC13559998; doi:10.1016/j.chpulm.2026.100263)
Supplement: Supplementary file 1 [file mmc1.docx]

**e-Table 1**: Serial Chest CT Follow-up (up to 2 years) of Patients with Non-Diagnostic Robotic Bronchoscopy Results

| Nodule Status | Values (n = 58) |
| --- | --- |
| Resolved/Smaller | 9 (15.5) |
| Stable | 11 (18.9) |
| False Negative | 13 (22.4) |
| Inflammation | 18 (31.0) |
| True Negative | 3 (5.17) |
| Lost To Follow-Up | 4 (6.90) |

**e-Table 2**: Demographic Features of Patients with Different Diagnoses

| Variables | All Patients  (n = 526) | Benign (no infection)  (n=65; 12.3%) | Benign (infection)  (n=107; 20.3%) | Cancer  (n = 296; 56.3%) | p-value |
| --- | --- | --- | --- | --- | --- |
|  |  |  |  |  |  |
| Age, years | 69 (61, 76) | 69 (63, 71) | 68 (57, 73) | 71 (64.5, 77) ^b, c^ | < 0.001 |
| Sex  Female  Male | 273 (51.9)  253 (48.1) | 35 (53.9)  30 (16.1) | 52 (49.6)  55 (51.4) | 153 (51.7)  143 (48.3) | 0.80 |
| BMI (kg/m^2) | 26.6 (22.8, 31.1) | 26.0 (23, 30.6) | 25.3 (21.7, 31.4) | 26.6 (22.8, 30.2) | 0.65 |
| Race  Caucasian  Hispanic  Black  Native American  Other | 424 (80.6)  38 (7.22)  22 (4.18)  4 (0.76)  38 (7.22) | 53 (81.5)  5 (7.69)  2 (3.08)  1 (1.54)  4 (6.15) | 80 (74.8)  6 (5.61)  5 (4.67)  2 (1.87)  14 (13.1) | 251 (84.8)  18 (6.08)  12 (4.05)  1 (0.34)  14 (4.73) | 0.11 |
| Smoking Status | 136 (25.9) | 16 (24.6) | 25 (23.4) | 87 (29.4) | 0.45 |
| Pack per year | 25 (0, 42) | 21.5 (0, 37) | 17 (0, 39) | 30 (10, 50) ^b, 3^ | < 0.001 |
| Quit in years | 9 (2, 23) | 2 (0.20, 15) | 8 (2.5, 22) | 10.5 (3, 26) | 0.16 |
| Cocci serology or history | 57 (10.8) | 0 (0.0) | 35 (32.7) ^a^ | 15 (5.07) ^c^ | < 0.001 |
| Multiple Biopsies | 150 (25.5) | 19 (29.2) | 25 (23.4) | 88 (29.7) | 0.45 |
| Bilateral (n = 150) | 82 (54.7) | 9 (47.4) | 13 (52.0) | 49 (55.7) | 0.78 |
|  |  |  |  |  |  |

Data presented as median and interquartile range (Q1,Q3) or no. (%). Continuous variables were compared using the Kruskal-Wallis test. Categorical variables were compared using chi-squared analysis or Fisher’s exact test.

^a^ Statistical difference between group 1 and group 2 following Bonferroni Correction (p<0.016)

^b^ Statistical difference between group 1 and group 3 following Bonferroni Correction (p<0.016)

^c^ Statistical difference between group 2 and group 3 following Bonferroni Correction (p<0.016)

Non diagnostic n=58 (not shown)

Abbreviation: BMI, body mass index.

**e-Table 3:** Nodule Size and Distribution in Patients with Different Diagnoses

|  | Infection  (n = 115; 26.1%) | Malignancy  (n = 261; 59.2%) | Benign  (no infections)  (n= 65; 14.7%) | p-value^1^ |
| --- | --- | --- | --- | --- |
| Number of Nodules | 144 | 355 | 92 |  |
| Nodule Size (mm)  0-5  6-10  11-20  21-30  >31 | 6 (4.17)  22 (15.3)  59 (40.9)  31 (21.5)  26 (18.1) | 11 (3.10)  61 (17.2)  148 (41.7)  83 (23.4)  52 (14.7) | 10 (10.9)  21 (22.8)  35 (38.0)  20 (21.7)  6 (6.52) | 0.004 |
| Nodule Location  inner 1/3rd  middle 1/3rd  outer 1/3rd | 19 (13.2)  40 (27.8)  85 (59.0) | 77 (21.7)  113 (31.8)  165 (46.5) | 18 (10.9)  35 (38.0)  47 (51.1) | < 0.001 |
|  |  |  |  |  |

P-values calculated using Multinomial logistic regression to associate nodule characteristics with cancer/infection status, adjusting for age, sex, race, and smoking status.

**e-Table 4**: Comparison of Infection Types between Positive Infection Status.

|  | Cocci in Serology | |  | Fungal Culture | | |  | Bacteria Culture | |  |
| --- | --- | --- | --- | --- | --- | --- | --- | --- | --- | --- |
| Infections | No  (n = 469) | Yes  (n = 57) | | No  (n = 465) | Yes  (n = 61) | | | No  (n = 471) | Yes  (n = 55) | |
|  |  |  | |  |  | | |  |  | |
| Cocci on Pathology | 13 (2.77) | 7 (11.3)** | | 13 (2.80) | 7 (11.5)*** | | | 18 (3.82) | 2 (3.64) | |
| Cocci in Culture | 4 (0.85) | 10 (17.5)*** | | 0 (0.0) | 14 (22.9)** | | | 12 (2.55) | 2 (3.64) | |
| Fungal Culture | 44 (9.38) | 17 (29.8)*** | | N/A | N/A | | | 41 (8.70) | 20 (36.4)*** | |
| Aspergillus Culture | 22 (4.69) | 6 (10.5) | | 0 (0.0) | 28 (45.9)*** | | | 19 (4.03) | 9 (16.4)** | |
| Other Fungus | 19 (4.05) | 3 (5.26) | | 0 (0.0) | 22 (36.1)*** | | | 13 (2.76) | 9 (16.4)*** | |
| AFB Culture | 13 (2.77) | 4 (7.02) | | 14 (3.01) | 3 (4.92) | | | 16 (3.40) | 1 (1.82) | |
| Bacteria Culture | 47 (10.0) | 8 (14.0) | | 35 (7.53) | 20 (32.8)*** | | | N/A | N/A | |
|  |  |  | |  |  | | |  |  | |
|  |  |  | |  |  | | |  |  | |
|  | AFB in Culture | |  | Aspergillus | |  | |  |  | |
|  | No  N = 509 | Yes  N = 17 | | No  N = 498 | Yes  N = 28 | | |  |  | |
|  |  |  | |  |  | | |  |  | |
| Cocci on Pathology | 20 (3.93) | 0 (0.0) | | 17 (3.41) | 3 (10.7) | | |  |  | |
| Cocci in Culture | 14 (2.75) | 0 (0.0) | | 12 (2.41) | 2 (7.14) | | |  |  | |
| Fungal Culture | 58 (11.4) | 3 (17.6) | | 33 (6.63) | 28 (100.0)*** | | |  |  | |
| Aspergillus Culture | 25 (4.91) | 3 (17.6) | | N/A | N/A | | |  |  | |
| Other Fungus | 22 (4.32) | 0 (0.0) | | 14 (2.81) | 3 (10.7) | | |  |  | |
| AFB Culture | N/A | N/A | | 21 (4.22) | 1 (3.57) | | |  |  | |
| Bacteria Culture | 54 (10.6) | 1 (5.88) | | 46 (9.24) | 9 (32.1)** | | |  |  | |
|  |  |  | |  |  | | |  |  | |

Categorical variables compared using Chi-Squared Analysis or Fisher’s Exact Test.

*p < 0.05, **p < 0.01, ***p < 0.001 between the groups.

**e-Table 5:** Type of Cancer and Nodule Characteristics in Patients with a Diagnosis of Malignancy and Infection.

| Variables | Malignancy with infection  (n = 35) |
| --- | --- |
| Type of Cancer  Squamous cell carcinoma  Adenocarcinoma  Neuroendocrine  Other | 6 (17.1)  22 (62.9)  2 (5.71)  5 (14.3) |
|  |  |
| Single vs Multiple nodules  Single  Multiple | 27 (77.1)  8 (22.9) |
|  |  |
| Nodule size (mm) (N = 46 nodules)  0-5  6-10  11-20  21-30  >31 | 2 (4.35)  7 (15.2)  20 (43.5)  7 (15.2)  10 (21.7) |
|  |  |
| Nodule location (N = 46 nodules)  Inner 1/3rd  Middle 1/3rd  Outer 1/3rd | 11 (23.9)  9 (19.6)  26 (56.5) |
|  |  |
| Type of infection* |  |
| Fungal Culture | 8 (22.9) |
| Aspergillus Culture | 3 (8.57) |
| Other Fungus | 5 (14.3) |
| AFB Culture | 1 (2.86) |
| Bacteria Culture | 14 (40.0) |
|  |  |
| Number of infections  1  2  3 | 26 (74.3)  4 (11.4)  5 (14.3) |
|  |  |

Data presented at no (%). Abbreviation: AFB, acid-fast bacteria.

**Supplemental Methods: Standardized Data Sheet**

Variables MRN:

DOP:

Age: years (continuous variable) at time of DOP

BMI: (continuous variable) find in Results review -> pharmacy view

Sex: (female 0; male 1)

Race: (Caucasian 0; Hispanic 1; black 2; native American 3 and other 4) Smoking: (no 0, yes 1)

Cigarette pack per year (continuous variable)

Quit in years (continuous variable)

Cocci serology or history (no 0, yes 1)

Number of nodule biopsy (this is from procedure note) 1= multiple, 0 = single nodule

Bronchoscopist: Khokar 0 and Saeed 1

Location of nodule: put 1 in the appropriate lobe

Location of nodules biopsied: 1= bilateral, 0= unilateral

Size of nodule: (0= 0-5 mm, 1= 6-10mm, 2= 11-20mm, 3= 21-30 mm and 4 = >31mm) Nodule location: follow the chart: distribution of pulmonary nodule

-	3 cm to the pleural edge is outer 1/3rd = 2

-	rest is middle 1/3rd = 1

-	3cm from the hilum is the inner 1/3rd = 0

Pathology diagnosis: cancer 1 benign 0

Bronchus sign (presence of airway in the nodule): 0= absent, 1= present

Radial signal (put one for the right option: absent 0, concentric 1, and eccentric 2) Intraoperative CT use (not used= 0, used= 1)

Fiducial placement: 1= yes and 0= no (verify in perioperative EN Intra-op note, sometimes not listed in procedure note)

Biopsy instrument: put 1 under instrument used

Bleeding (1= yes) for bleeding volume: >25 mL =0 and >50 mL =1; bleeding which required management and procedure was stopped

Procedure pneumo: 1= yes and 0= no (document chest tube vs observation after pneumo) (reason for admission; don’t go by radiology impression)

Hospitalization yes= 1, no= 0

Bronchoscopy time in min from nursing documentation (scope in to out)

Total in room time: from nursing documentation in min; look into perioperative EN Intra-op note

Pathologist: (0= Hahn, 1= Feitz, 2= Hesham, 3= Gomez, 4= Daniel, 5 others)

Preliminary: (0= granuloma, 1= cancer, 2= non-diagnostic, 3 other: inflammation/ organizing pneumonia etc)

Final: (0= granuloma, 1= cancer, 2= non- diagnostic, 3= other)

Concordance (0=no and 1=yes) [Prelim and final reports were same or different?]

Pathology put 1 if positive

BAL culture, put 1 if positive

Follow up CT chest: this is for benign diagnosis and chronic inflammation (resolved =0 and still present

=1)
